# Molecular docking of polyphenols and screening of antioxidant and anticancer activity of *Artemisia monosperma* leaf extracts in human cancer cells

**DOI:** 10.1038/s41598-026-49276-7

**Published:** 2026-05-02

**Authors:** Mosaad A. Abdel-Wahhab, Zeinab A. El-Shahid, Zeinab K. Hamza, Eslam R. El-Sawy, Aziza A. El-Nekeety, Sekena H. Abdel-Aziem, Hagar E. Mohammed

**Affiliations:** 1 Food Toxicology & Contaminants Department, National Research Centre, Dokki, Cairo, Egypt; 2 Chemistry of Natural & Microbial Products Department, National Research Centre, Dokki, Cairo Egypt; 3 Chemistry of Natural Compounds Department, National Research Centre, Dokki, Cairo, Egypt; 4 Cell Biology Department, National Research Centre, Dokki, Cairo Egypt; 5 Zoology Department, Faculty of Science, Arish University, North Sinai, Egypt

**Keywords:** *Artemisia monosperma*, Antioxidant, Anticancer, Molecular docking, Anti-apoptotic proteins, Gene expression, Biochemistry, Biotechnology, Cancer, Cell biology, Drug discovery, Plant sciences

## Abstract

This study evaluated the polyphenol content of leaf extracts from *Artemisia monosperma* (AM) and investigated their antioxidant properties, cytotoxic effects, and potential to induce DNA damage in human cancer cell lines. High-performance liquid chromatography (HPLC) quantified polyphenols in methanolic (AMM), ethanolic (AME), and aqueous (AMA) extracts, identifying 13 compounds in AME and 12 in AMA. AMM exhibited the strongest antioxidant activity (IC_50_ = 24 µg/ml). Both AME and AMM demonstrated potent anticancer activity against HCT-116 (IC₅₀ = 0.38 µg/mL for AMM) and HUH-7 (IC₅₀ = 21.95 µg/mL for AMM) cells, while exhibiting minimal cytotoxicity toward normal skin fibroblast cells (BJ-1; IC₅₀ = 13.05 µg/mL for AMM), with AMM demonstrating particular selectivity for HCT-116 cells. AMM induced DNA fragmentation and modulated apoptosis-related gene expression (*Bax*,* Bcl-2*,* p53*) in HUH-7 cells and caused cell cycle arrest at G0/G1 phase in HCT-116 cells. Molecular docking further supported AMM’s apoptosis activity. These results position *A. monosperma* as a rich source of bioactive polyphenols and antioxidants, with AMM showing promise as a therapeutic agent, especially for colorectal cancer.

**Supplementary Information:**

The online version contains supplementary material available at 10.1038/s41598-026-49276-7.

## Introduction

*Artemisia (A)* is an aromatic medicinal plant exceedingly used for the treatment of various illnesses in traditional medicine by several cultures^1^. The products of A. Abla Asso were used by ancient Egyptians as a pest repellent^2^. *A. annua* L was used in Africa and Asia several years ago for fever and malaria treatment in the form of pressed juice or tea^3^. It was officially listed by Pharmacopeia of China as a remedy for malaria and fever, at a 4.5–9 g daily dose of dried herb as an infusion preparation for clinical trials^4^. *A. annua* L is effective as anti-plasmodial, anti-hyperlipidemic, anti-inflammatory, anti-convulsant, anti-cholesterolemic, anti-microbial, and antiviral^5^. It has also shown potent pharmacological activities, including anti-tumor, anti-obesity, and anti-inflammatory^6^. This plant is effective for the treatment of inflammatory disorders such as coughs due to colds, diarrhea, bronchitis, infectious diseases such as scabies, syphilis, and skin diseases, among other different biological properties^7^. In Egypt, *Artemisia Judaica* L. is known in Arabic as ‘shih balady’ and is considered an herbal medicine rich in camphor and piperitone^8^.

Previous reports showed that Artemisinin, the main bioactive compound in *Artemisia*^9^, is effective against different cancer cell lines, and numerous tumor types, including human leukemia, breast cancer, colon cancer, small-cell lung carcinomas, and hepatitis B^10^. This plant also showed beneficial activities in the treatment of widely spread diseases such as diabetes, obesity, aging-related disorders^11^, and malaria treatment^12^. Plant materials, including extracts, and essential oils, are used in folk medicine because of their unique activities and their high content of bioactive ingredients^13^. However, obvious differences were observed in the health benefits and chemical compositions of *Artemisia spp*. according to the geographical location^14^. There is scientific evidence to support the fact that the chemical composition of plants varies due to climatic conditions and geographic locations^15^, the plant material used, and the treatment method, unlike the essential oil composition, which may vary slightly^16^.

Hepatocellular carcinoma (HCC) is considered the 6th most common type of cancer worldwide^17^. This type of cancer is represented as the 4th most common cancer in Egypt^18^. On the other hand, colorectal cancer (CRC) remains the third most common cancer in recent years. It is the second leading global cause of cancer death due to the increased incidence and the absence of screening programs and treatment strategies^19^. Oxidative stress and generation of reactive oxygen species (ROS) are the main causes of several types of cancer^20^. ROS are important for cancer cell homeostasis and are involved in the development of cellular processes such as proliferation, migration, differentiation, and cell death. On the contrary, high levels of ROS are harmful to cancer cells and eventually lead to cell death^21^. ROS interact with cellular macromolecules such as proteins and DNA and interfere with cellular signaling pathways, including transport mechanisms and protein kinases^22^. ROS are produced continuously as byproducts of oxidative phosphorylation, or mitochondrial electron transfer in aerobic respiration, and are maintained by enzymatic antioxidants in homeostasis^23^. Endogenous enzymatic or non-enzymatic antioxidants such as glutathione (GSH), catalase (CAT), and SOD, as well as exogenous antioxidants such as polyphenols, vitamins (C and E), or carotenoids found in the diet, work together to maintain cellular redox homeostasis^24^. These antioxidants act as protective agents against cancer as they can neutralize ROS-induced DNA damage, which leads to cancer^25^.

Molecular docking (MD) is a computational biology and bioinformatics procedure that supports the in vitro and in vivo evaluation of the anticancer potency of natural compounds. This procedure is used to model the interaction between natural compounds and target proteins^26^. MD has grown to be a significant step in the drug discovery process^27^. Moreover, MD can be applied as a simulated drug screening technique based on the 3D crystal structures of target proteins to predict binding protein interactions^28^. Bioinformatics calculations are based on parameters that affect the attraction and repulsion between the target and the compound, for example, hydrogen bonds, van der Waals interactions, covalent bonds, unfavorable donor-donor interactions, and hydrophobic interactions. To ensure the safety of these leaf extracts, this study aimed to (1) determine the polyphenols in the aqueous, ethanolic, and methanolic leaf extracts of *A. monosperma* (AMA, MAE, and AMM, respectively), (2) evaluate the antioxidant and anticancer activity against hepatocellular carcinoma (HUH-7), human colorectal carcinoma (HCT-116) cell lines, and normal human fibroblast (BJ-1), and (3) evaluate the expression of apoptotic genes and DNA damage in the cancer cell lines. Additionally, molecular docking of the primary bioactive ingredients in AMM against the binding site of the anti-apoptotic proteins Bcl-2 and p53 was performed.

## Results

### HPLC analysis of the polyphenols

**Fig. 1:**
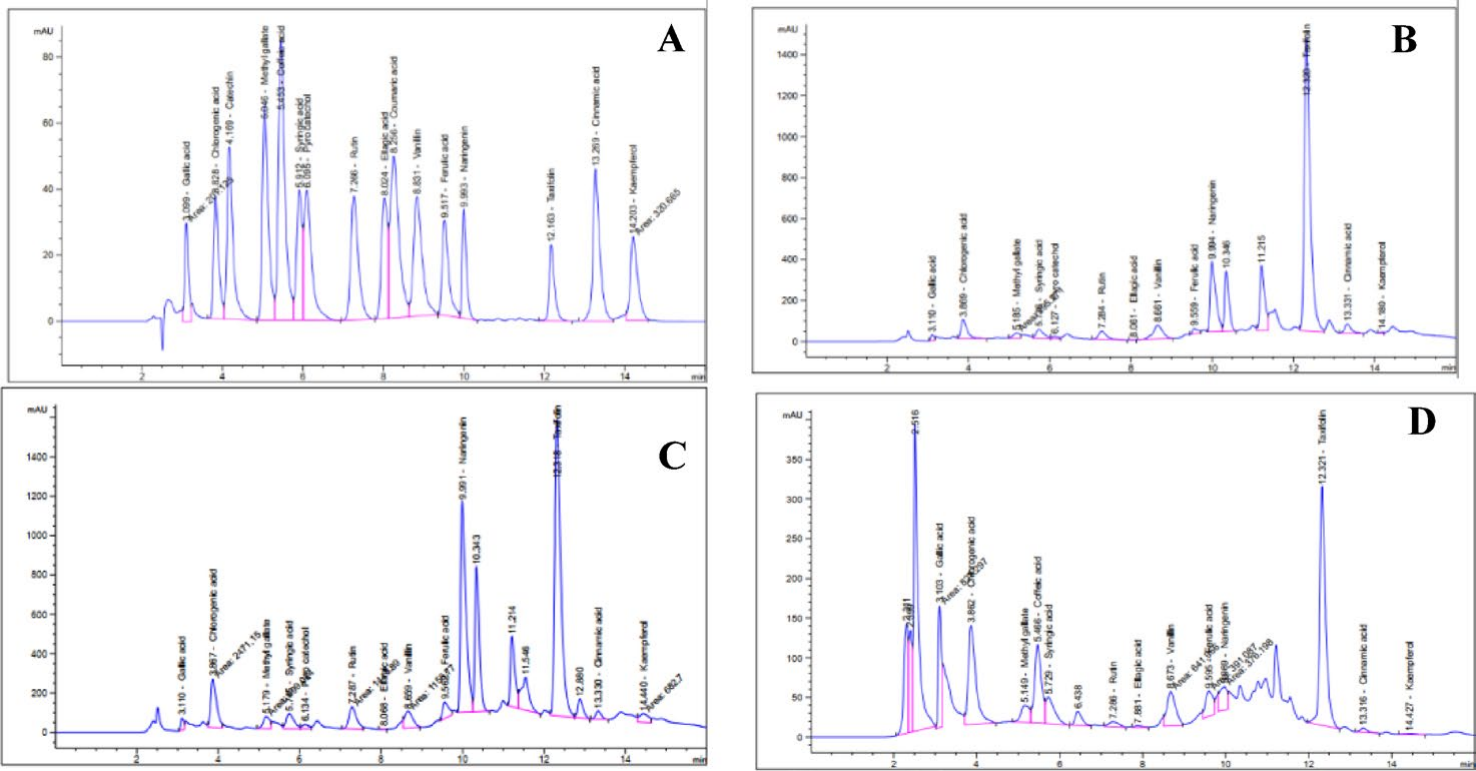
HPLC chromatograms for **(A)** standards polyphenols, **(B)** AMM, **(C)** AME and **(D)** AMA extracts *A*. *monosperma* leaf.

The HPLC analysis identified 13 compounds in the AMM and AME; however, only 12 compounds were identified in the AMA (Table 1; Fig. 1). Kaempferol, taxifolin, naringenin, gallic acid, chlorogenic acid, caffeic acid, and vanillin were the most abundant polyphenols in the AMM, with concentrations of 233.75, 201.38, 96.27, 67.00, 55.81, 34.06, and 33.90%, respectively. Taxifolin, gallic acid, chlorogenic acid, naringenin, kaempferol, and caffeic acid were the most abundant components in the AME, with concentrations of 152.31, 106.72, 62.40, 60.02, 36.37, and 33.31%, respectively. On the other hand, the HPLC analysis of the AMA showed that the main constituents were gallic acid, chlorogenic acid, and taxifolin, with concentrations of 145.48, 70.93, and 34.99, respectively. Additionally, cinnamic acid was completely absent in the AMM, and catechin was absent in the AME, while catechin and caffeic acid were absent in the AMA.

**Table 1:** HPLC analysis of the total polyphenols of the three extracts of A. monosperma*.

polyphenols	Concentration (µg/ml)		
	AMM	AME	AMA
Gallic acid	67.00	106.72	145.48
Chlorogenic acid	55.81	62.40	70.83
Catechin	18.21	0.00	0.00
Methyl gallate	16.41	17.74	10.16
Coffeic acid	34.06	33.31	0.00
Syringic acid	15.43	10.01	14.29
Rutin	7.47	12.23	8.09
Coumaric acid	4.95	7.60	3.52
Vanillin	33.90	29.66	2.15
Ferulic acid	8.59	2.87	2.63
Naringenin	96.27	60.02	20.39
Taxifolin	201.38	152.31	34.99
Cinnamic acid	0.00	16.97	9.23
Kaempferol	233.75	36.37	10.12
Total	793.23	548.21	331.88

* The data represent absolute concentrations obtained from quantifying individual compounds via calibration curves prepared with authentic standards.

### Antioxidant activity

**Fig. 2:**
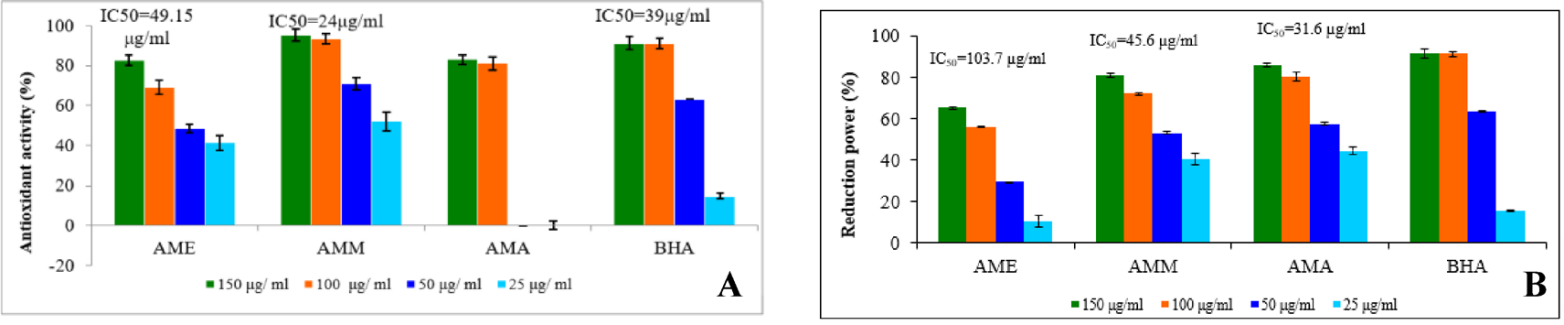
Total antioxidant activity of *A*. *monosperma* leaf extracts using **(a)** DPPH radical Scavenging activity and **(b)** Ferric Reducing Antioxidant Power (FRAP).

The results of DPPH assay revealed that the scavenging activities of AME, AMM, and AMA of *A. monosperma* were 82.67 ± 0.43, 95.48 ± 0.02, and 83 ± 0.09%, respectively at a concentration of 150 µg/ml (Fig. 2A), whereas, it was 91.23 ± 0.08 for the standard BHA. AMA showed the lowest antioxidant activity against DPPH compared with the standard BHA, whereas the IC_50_ values for AME was 49.5 µg/ml. The AMM showed strong scavenging activity with IC_50_ of 24 µg/ml compared to BHA (IC_50_ of 39 µg/ml). These results indicated that the DPPH radical scavenging activity was positively correlated to the concentration of the extract. Moreover, the results of the FRAP assay revealed that AMA showed the greatest ferric-reducing power (86%), followed by AMM (81%); at a concentration of 150 µg/ml; whereas, AME exhibited the lowest activity (65%) in reducing ferric ion. AMM possesses a significant reducing capacity ranging from 81–40 µg/ml in a dose-dependent manner with IC_50_ 45.6 µg/ml. In contrast, the AMA exhibited higher capacity in reducing ferric ion (Fe^3+^) to ferrous ion (Fe^2+^) with IC_50_ of 31.6 µg/ml (Fig. 2B).

### Cytotoxic activity

**Fig. 3:**
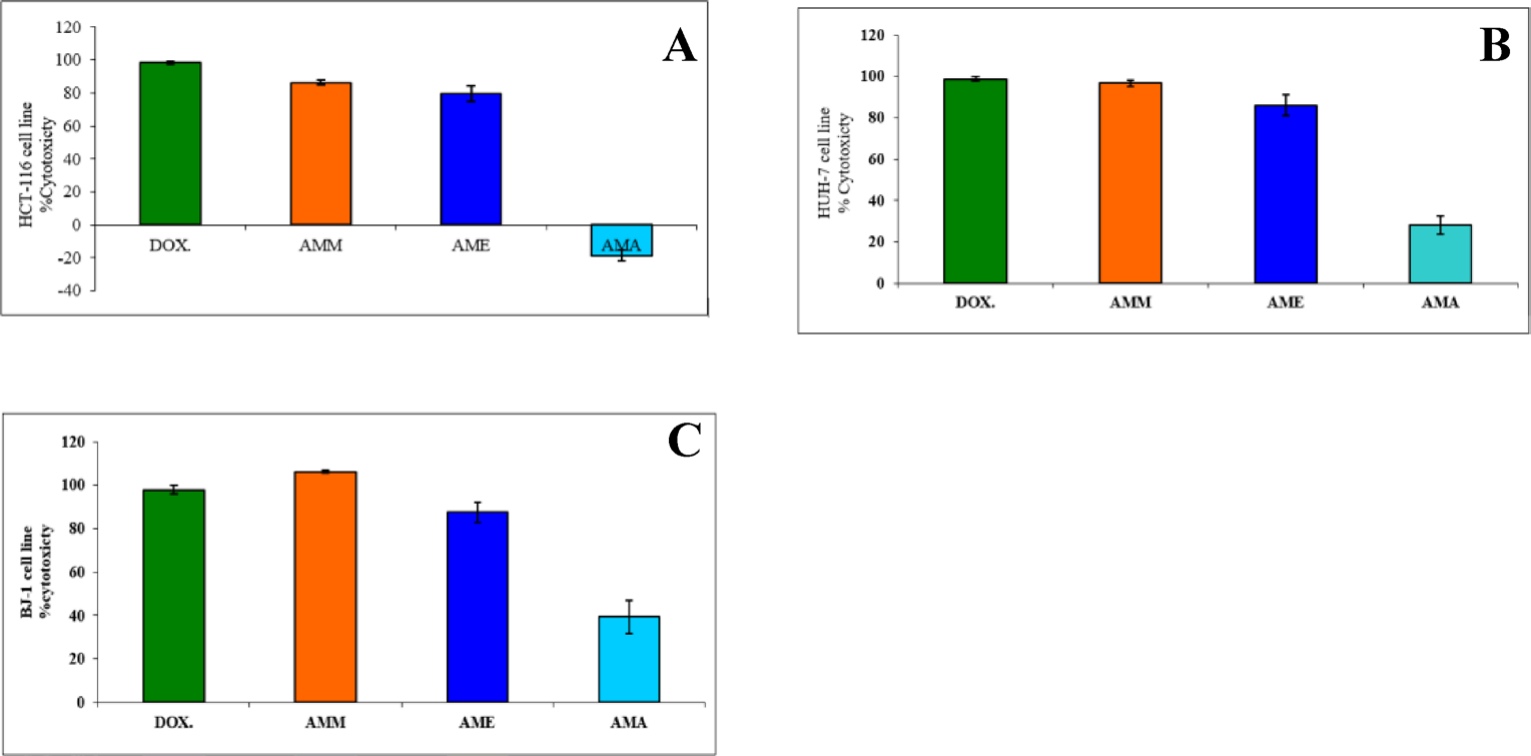
Average % cytotoxicity of three *A*. *monosperma* leaf extracts and doxorubicin (DOX) against: **(A)** HCT-116, **(B)** HUH-7 and **(C)** BJ-1 normal cell lines.

The three *A. monosperma* leaf extracts were screened for their in vitro cytotoxic activities using an MTT assay on a monolayer 2D model. The tested extracts were demonstrated to have variable anticancer activities towards the tested cell lines (Fig. 3). Both AMM and AME showed potent cytotoxic effects against the HCT-116 cell line reached 79.8, and 78.57%, respectively compared to DOX which recorded 98.4%; however, AMA showed a weak cytotoxic effect (Fig. 3A). The cytotoxic activity of AMM, AME, and AMA in HUH-7 cells (Fig. 3B) recorded 96.76, 86.02, and 28.10% respectively, compared to the positive control doxorubicin (98.7%). However, the cytotoxic effect of the three extracts against the BJ-1 cell line recorded 106.2, 87.4, and 39.33% for AMM, AME, and AMA, respectively, compared with DOX which recorded 97.8% (Fig. 3C). The IC_50_ for the three extracts and DOX revealed that AMM and AME showed the most potent and most selective against HUH-7 cells and the recorded IC_50_ were 21.95 and 71.12 µg/ml, respectively (Table 2). However, AMM showed the most potent cytotoxic activity against HCT-116 with an IC_50_ value of 0.38 µg/ml. However, IC_50_ for AME against HCT-116 cell line was 49.87 µg/ml, whereas AMA showed low cytotoxic activity (< 60%). These results demonstrated that AMM exhibited the greatest selectivity amongst all *A. monosperma* leaf extracts towards HCT-116 cells with SI values of > 34 (Table 3).

**Table 2:** Inhibitory concentration 50 (IC_50_) of the three *A. monosperma* leaf extracts against HUH-7, HCT-116, and BJ-1 cell lines.

Cell line Extracts	HUH-7	HCT-116	BJ-1
	IC_50_ (µg/ml)	IC_50_ (µg/ml)	IC_50_ (µg/ml)
DOX	2.0 ± 0.18	2.2 ± 0.10	13.5 ± 0.60
AMM	21.95 ± 0.80	0.38 ± 0.03	13.05 ± 0.09
AME	71.12 ± 1.05	49.87 ± 0.12	50.66 ± 0.12
AMA	--	--	159.6 ± 0.34

IC_50_ values are the mean ± SD of three separate experiments (µg/ml).

**Table 3:** Selectivity index (SI) of the active cytotoxic three *A. monosperma* leaf extracts against HCT-116 and HUH-7.

Cell line Extract	HCT-116	HUH-7
AMM	≥ 34.5	≥ 0.59
AME	≥ 1.02	≥ 0.71
AMA	---	---

### Apoptosis-related gene expression

**Fig. 4:**
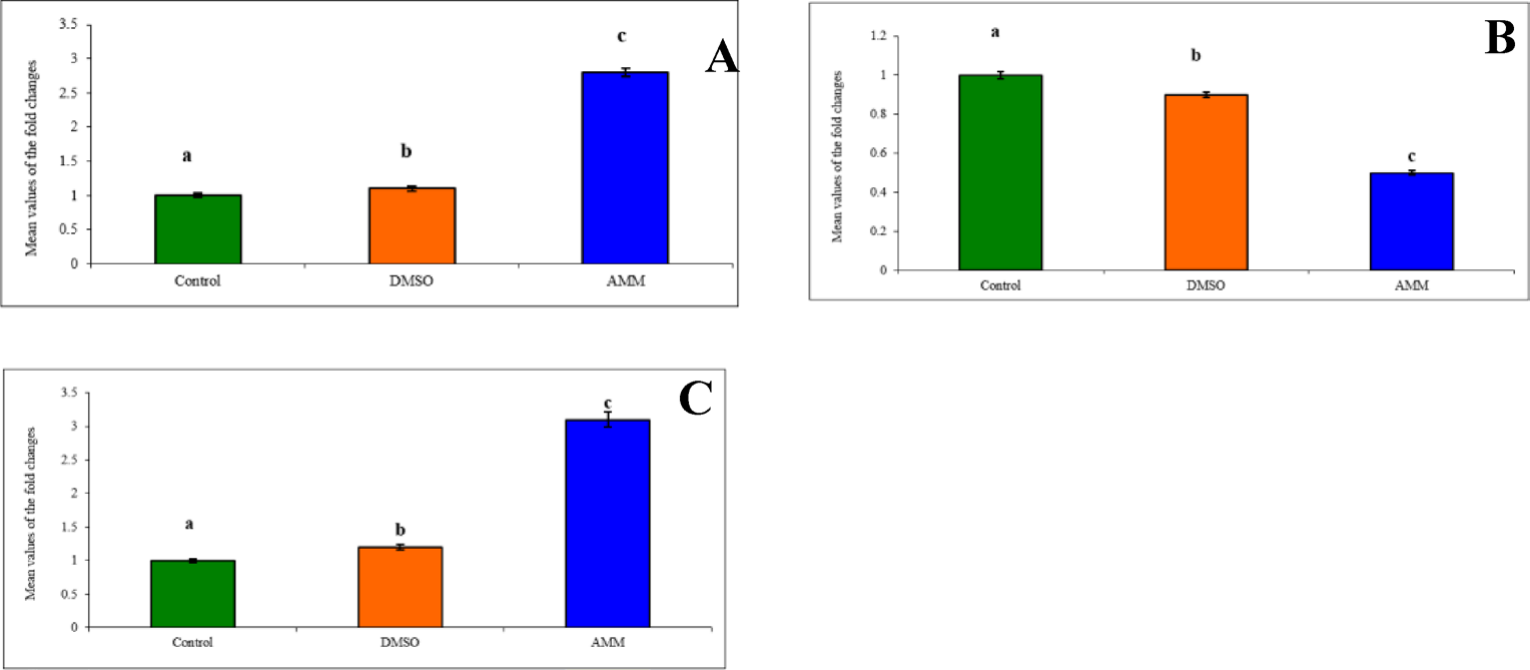
The expression level of apoptosis-related gene **(A)** *BAX*, **(B)** *Bcl-2* and **(C)** *p53* in HCT-116 human colorectal carcinoma cell lines treated with AMM (0.38 µg/ml for 24 h). Column superscripts with different letter are significantly different at *P* ≤ 0.05.

The IC_50_ of AMM (0.38 µg/ml) was used to evaluate the expression levels of Bax, Bcl-2, and p53 mRNAs in HCT-116 cell lines using SYBR Green real-time quantitative PCR, compared to the untreated control cell line. The results showed that the expression of Bax mRNA was increased by 2.8-fold compared to the control untreated cells (Fig. 4A). The expression of Bcl-2 mRNA was decreased by 0.5-fold (Fig. 4B), whereas, p53 mRNA expression was increased 3.1-fold compared to the control cells (Fig. 4C). These results indicated that the AMM killed the HCT-116 cell lines mostly through apoptotic mechanisms involving these genes.

**Fig. 5:**
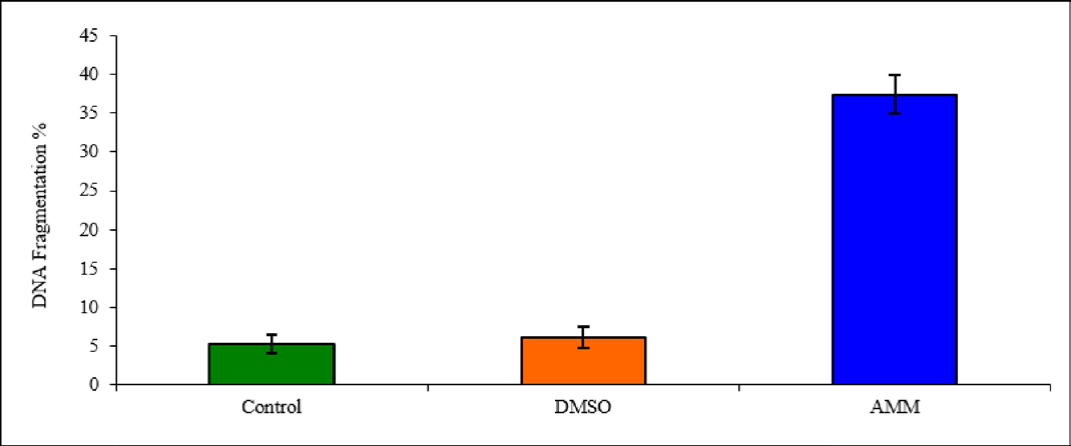
Quantitative estimation of DNA fragmentation by diphenylamine (DPA) assay in HCT-116 cell lines treated with AMM and untreated cells. Values are represented as mean ± SD and the probability of significant was based on *p* < 0.001 vs. control.

The effect of the AMM on DNA damage in HCT-116 cell lines was assessed using the DPA assay to measure the relative quantity of DNA fragments in the treated cells. The results indicated that this extract induced DNA damage, with a higher proportion of fragmentation (37.47%) than in the untreated control cells (5.27%), or DMSO-treated cells (6.11%) as shown in Fig. 5. The DNA damage caused by extract in HCT-116 cell lines was further examined using gel electrophoresis, which detects evident DNA seamier. The findings of this assay revealed that no such DNA smear was observed in the untreated control cells and the smear appeared as clear bands of intact DNA (undamaged), indicating no DNA damage at all, as shown in Fig. (1 S, supplementary file). DNA fragmentation is believed to be a hallmark of the apoptotic process that begins within the cell, further confirming that AMM caused cell death by activating the apoptosis mechanism.

### Cell cycle arrest

**Fig. 6:**
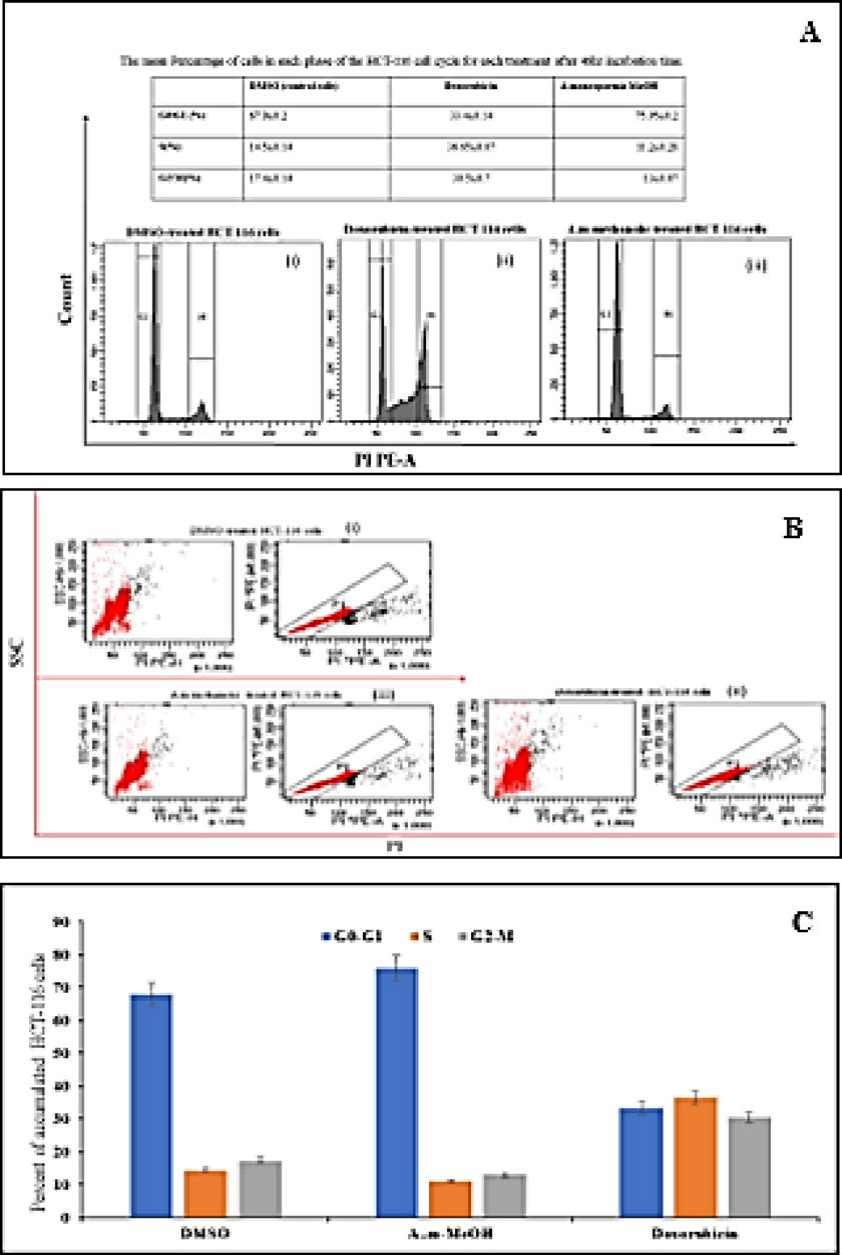
The impact of AMM extract at its IC_50_ concentration and doxorubicin (used as a positive control) on the cell cycle distribution of HCT-116 cells after 48 h of treatment was analyzed by flow cytometry. **(A)** Comparison of cell cycle distribution between untreated control HCT-116 cells and those treated with the extract; **(B)** Dot plots showing apoptotic cell populations stained with PI/PE for cell cycle analysis; and **(C)** A column chart illustrating the cell cycle distribution of HCT-116 cells. HCT-116 cells were seeded at a density of 1 × 10^6 cells per well in a 6-well plate, treated with the IC_50_ concentration, and incubated at 37 °C for 48 h. PI: propidium Iodide stain, SSC: side scatter.

The cell cycle arrest results demonstrated that HCT-116 colon cancer cells treated with AMM extract (Fig. 6A, B) exhibited an increased percentage of cells in the G0/G1 phase, indicated by a pronounced sharp peak, compared to the DMSO-treated negative control. In contrast, cells treated with doxorubicin underwent cell cycle arrest at the G2/M phase. Moreover, the cell cycle analysis of HCT-116 cells treated with AMM extract showed a significant accumulation in the G0/G1 phase when plated at a density of 1 × 10^6 cells per well in a 6-well plate (Fig. 6C).

### Molecular docking

The induction of apoptosis by the major bioactive constituents in AMM promoted us to investigate the interactions with the target anti-apoptotic proteins *Bcl-2* and *p53* using molecular docking. Accordingly, the apoptotic protein structure was derived from the PDB database. Their native ligand fundamental poses served as references to evaluate the simulation procedure and set the dimensions and positioning of the grid box. It was noticed that the native ligand (*IL0*) of the target anti-apoptotic protein *Bcl-2* (PDB ID: 2O2F) exhibits multiple hydrogen bonds interaction with the target, including ASP108, GLY142, and TRP141, in addition to Pi-Pi and Pi-alkyl interactions (Table S1, Fig. 2S, supplementary file). Regarding the target anti-apoptotic protein *p53* (PDB: 1TTV), the natural ligand (IMY) showed Pi-Pi interactions (Table S1, Fig. 3S, supplementary file).

### Assessment of the binding mode of the polyphenolic compounds with Bcl-2 (PDB ID: 2O2F)

**Fig. 7:**
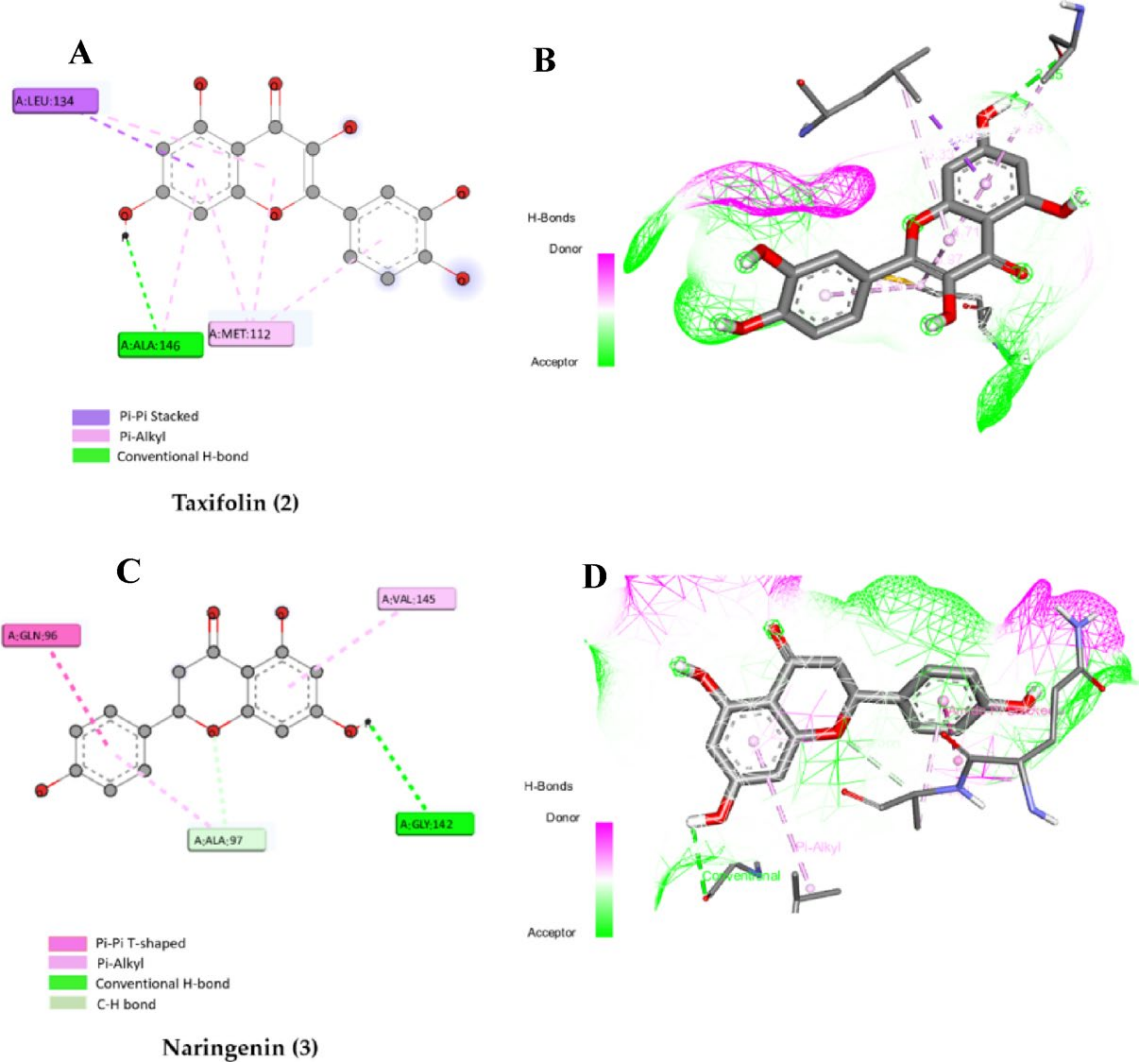
(A). The 2D binding mode of taxifolin (2) within the binding pocket of *Bcl-2* (PDB ID: 2O2F), (**B)**. The 3D binding mode of taxifolin (2) within the binding pocket of *Bcl-2* (PDB: 2O2F), (**C) A.** The 2D binding mode of naringenin (3) within the binding pocket of *Bcl-2* (PDB ID: 2O2F), and **(D)** The 3D binding mode of naringenin (3) within the binding pocket of *Bcl-2* (PDB: 2O2F).

The docking results of all examined polyphenols indicated that all of them were superimposable, in addition, they showed a good binding mechanism with the active pocket by forming numerous interactions with the key amino acids of *Bcl-2* similarly as LI0 (Table S1). Notably, among the seven compounds examined, taxifolin (2) and naringenin (3) showed the closest binding energy in comparison to the native ligand LI0 with values of −6.9 kcal/mol (Table 4; Figs. 7A, B).

**Table 4:** The molecular docking result of the major bioactive constituents of AMM extract against the target anti-apoptotic proteins Bcl-2 (PDB ID: 2O2F) and p53 (PDB: 1TTV).

Target	PDB ID	Score Kcal/mol							
*Bcl-2*	2O2F	**LI0**	**1**	**2**	**3**	**4**	**5**	**6**	**7**
		−10.3	−6.7	−6.9	−6.9	−5.4	−5.5	−6.7	−5.7
*p53*	1TTV	**IMY**	**1**	**2**	**3**	**4**	**5**	**6**	**7**
		−7.5	−6.5	−5.8	−6.3	−4.5	−5.2	−5.1	−5.1

LI0: native ligand of *Bcl-2* (PDB ID: 2O2F); IMY: native ligand of *p53* (PDB ID: 1TTV), 1: Kaempferol, 2: taxifolin, 3: naringenin, 4: gallic acid, 5: chlorogenic acid, 6: coffeic acid, 7: vanillin.

### Assessment of the binding mode of the polyphenolic compounds with p53 (PDB ID: 1TTV)

**Fig. 8:**
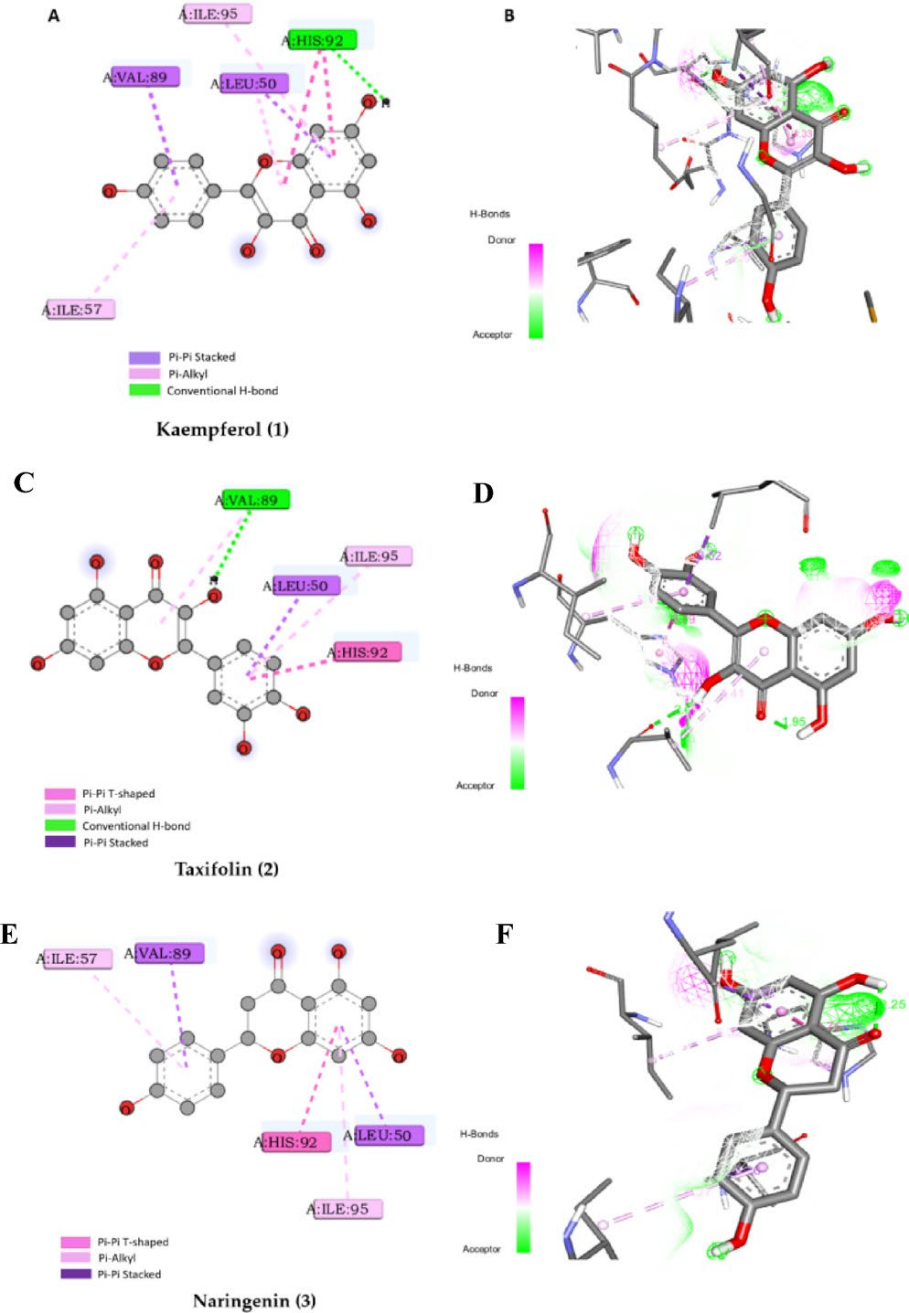
**(A)** The 2D binding mode of kaempferol (1) within the binding pocket of *P53* (PDB ID: 1TTV), (**B**) The 3D binding mode of kaempferol (1) within the binding pocket of *p53* (PDB ID: 1TTV), **(C)** the 2D binding mode of taxifolin (2) within the binding pocket of *p53* (PDB ID: 1TTV). (**D)** the 3D binding mode of taxifolin (2) within the binding pocket of *p53* (PDB ID: 1TTV), **(E)**, the 2D binding mode of naringenin (3) within the binding pocket of *p53* (PDB ID: 1TTV), and (**F)** The 3D binding mode of naringenin (3) within the binding pocket of *p53* (PDB ID: 1TTV).

The docking result indicated that polyphenol compounds were adaptive to the active pocket of the *p53* protein. Interestingly, they showed a superior binding mechanism with the functional pocket by generating numerous interactions with key amino acids of *p53* compared to IMY, a native ligand, (Table S1). Among the seven compounds studied, kaempferol (1), taxifolin (2), and naringenin (3) showed the closest binding energy in comparison to IMY (−7.5 kcal/mol) with values of −6.5, −5.8, and − 6.3 kcal/mol, respectively (Table 4; Figs. 8A-F).

## Discussion

It has been reported that the extraction solvent has a substantial effect on the polyphenol content of plants^29^. Hence, optimizing sample preparation is critical for accurately analyzing distinct classes of phenolic metabolites in different foods. The HPLC analysis revealed that total polyphenols in the AMM, AME, and AMA were 793.23, 548.21, and 331.88 µg/ml, respectively. This difference is most likely due to the complex synthesis of some phenolic compounds that are soluble in methanol and ethanol and/or have high molecular weights compared to those soluble in the AMA^30^. In general, the type and polarity of the extraction solvent play an important role in the polyphenol content because the polarities of polyphenols are either polar or non-polar, and the optimum amount is obtained when the polar solvents are used^31^. This is owing to the interaction of the polar site in the antioxidant compounds (hydrogen bands) with the polar solvent as opposed to the non-polar ones^32^. Hence, the choice of an effective extraction solvent for phenolic compounds depends on the type of food matrix^33^. The HPLC results revealed that the AMM contained kaempferol, taxifolin, naringenin, gallic acid, chlorogenic acid, caffeic acid, and vanillin; whereas, the AME extract contained taxifolin, gallic acid, chlorogenic acid, naringenin, kaempferol, and caffeic acid. Meanwhile, the AMA was rich in gallic acid, chlorogenic acid, and taxifolin. These results are consistent with those reported by Carvalho et al.^34^ who found that ferulic acid conjugates and caffeic acid were the most common hydroxycinnamic acids in different species of *Artemisia*. Moreover, *Artemisia* spp has been reported to be rich in flavonoids and phenolic acids with caffeic acid dimer being particularly plentiful in this plant during the flowering stage^35,36^. On the other hand, Tan et al.^37^ found quite opposite results, reporting that the level of phenolic acids was lower than the flavonoids. The discrepancies in these results could be attributed to the season, plant harvesting stage^38^, and/or the cultivation temperatures^39^. Generally, polyphenol content exhibits considerable variability due to genetic, environmental, developmental (e.g., plant age), tissue-specific (e.g., leaves vs. fruits), and post-harvest factors (e.g., processing and storage).

The antioxidant activity of the leaf extracts was determined using DPPH radical scavenging activity and FRAP assays. In the DPPH assay, the 2,2-diphenyl-2-picrylhydrazyl hydrate (a nitrogen radical with maximal absorption at 517 nm) is converted to 1,1, diphenyl-2-picrylhydrazine when interacting with hydrogen donor species^40^, such as the antioxidants present in the sample, primarily the polyphenols^41^. This ability to donate hydrogen leads to the formation of a stable complex of free radicals, which terminates lipid peroxidation^42^. These results confirmed that the AMM exhibited strong DPPH radical scavenging activity followed by the AME and subsequently the AMA. However, in FRAP assay, the presence of reducers such as polyphenols in the leaf extracts causes the transformation of the Fe^+ 3^-TPTZ complex to blue-colored Fe^+ 2^-TPTZ via electron donation^43^. Taken together, the high antioxidant activity (as assessed by FRAP and DPPH assays) can be attributed to the synergistic effect of the various polyphenols present in the leaf extracts. These results are consistent with those reported previously which concluded that that polyphenols are the main contributor to antioxidant capacity^44^. The results showed that the AMM contains more antioxidant constituents than the AME or AMA. Thus, methanol is a potent solvent for optimizing the antioxidant compounds in the plant and agrees with those reported previously which indicated that the methanolic extract is the most efficient antioxidant in several *Artemisia* species^45,46^. Additionally, the antioxidant capacity of the AMM and AME reported herein was greater than those reported previously^47^.

The results also showed that the three tested leaf extracts had varying anticancer activity against HCT-116, HUH-7, and BJ-1 cell lines, with the AMM and AME exhibiting strong cytotoxicity against HUH-7 cells reaching 96, and 86%, respectively compared to the positive control doxorubicin (98.7%). However, the AMA demonstrated poor cytotoxic activity (< 60%), suggesting that the AMM and AME are powerful and selective against HUH-7 cells with IC_50_ values of 21.95 and 71.12, respectively. Additionally, these extracts exhibited high cytotoxicity against HCT-116 cancer cells reaching 79.8 and 78.57%, with an IC_50_s of 0.38, and 49.87 µg/ml, respectively. A previous report indicated that the methanolic extract showed strong cytotoxicity against the HCT-16 cell line, whereas, the aqueous extract was not cytotoxic against normal BJ-1 cells. Similar to these findings, Solowey et al.^48^ reported that *Artemisia* exhibited high cytotoxicity against various cancer cell lines but did not cause any cytotoxicity to normal human cells. Additionally, Lian et al.^49^ found that the methanolic extract of *Artemisia* had marked cytotoxicity and anti-proliferative effects against HCT-15, suppressing migratory potential and colony formation at an IC_50_ value of 50 µg/ml. Furthermore, Farshuri et al.^50^ found that hexane, chloroform, butanol, and aqueous extracts of *A. monosperma* reduced HCT-116 cell viability and altered cellular morphology in a dose-dependent way. These researchers confirmed that chloroform extract had a higher cytotoxic action, accelerated apoptosis, increased ROS generation, mitochondrial dysfunction, and activation of apoptosis receptor-related genes (*Bax*, *p53*, *caspase-3*, and *9*), as well as antiapoptotic genes (*Bcl-2*).

Although we only determined the polyphenols in the three leaf extracts, artemisinin is a well-known bioactive constituent of the extract that also plays an essential role in anticancer activity^51,52^. For instance, *Artemisia* is rich in artemisinin and its derivatives which have been shown to inhibit HUH-7 cell proliferation by blocking the *mTOR* and PI3K/AKT signaling pathways in HCC cell lines, regulate both angiogenesis and apoptosis in HCC, and control the proliferation and angiogenic potential of HCC cells^53^. These compounds also were found to down-regulate apoptosis and anti-apoptotic proteins *XIAP* and survive while boosting the expression of cleaved *caspase-3* and *PARP* resulting in increased HUH-7 apoptosis^54^. Additionally, the methanolic extract has been shown to efficiently reduce *STAT3* activation, resulting in decreased HCC cell proliferation and migration^51^.

Moreover, the AMM had the strongest anticancer activity against HCT-16 cell lines, therefore these cell lines were employed to assess the influence of this extract on the mRNA expression of *Bax*,* Bcl-2*, and *p53*. The results showed that the AMM increased the expression of *Bax* mRNA by 2.8-fold while decreasing the expression of *Bcl-2* mRNA by 0.5-fold and *p53* mRNA by 3.1-fold compared to the control cells. The findings suggested that AMM killed the HCT-116 cell lines mostly through the apoptosis mechanism mainly via these genes. Similar results were reported by Nazeri et al.^55^ who showed that the cytotoxic activities of the methanolic extract suppressed the growth of HCT-116 cells through prompting apoptosis as indicated by the activation of *caspase-3* and *Bcl-2*, up-regulation of *Bax*, and diminish MMP. Generally, AMM induced apoptotic cell death by increasing *Bax* mRNA expression while down-regulating *p53* and *Bcl-2* expression, which is widely established as a major regulator of outer mitochondrial membrane permeability. Moreover, the interaction of the anti-apoptotic protein, *Bcl-2* with the pro-apoptotic protein *Bax* promotes mitochondrial membrane loss^56^. Hence, the reduction of *Bcl-2* mRNA expression and elevation of *Bax* mRNA are vital in promoting apoptosis^57^. The strong anticancer effect is due to the higher content of polyphenols in the extract which increased the expression of apoptosis-related genes such as *caspase-8*,* −9*,* −3*, *Bcl-2*,* PARP*, *TNF* receptor, IkB, and NF-kB resulting in the down-regulation of anti-apoptotic genes and the decrease in NF-kB nuclear translocation. Hence, the polyphenols activate the mitochondrial intrinsic and *FAS*-mediated extrinsic apoptotic pathways^58^.

The effect of AMM on DNA fragmentation in HCT-116 cell lines was evaluated by the DPA assay, and the DNA damage was assessed by gel electrophoresis. The induction of the DNA repair system is considered one of the mechanisms used to evaluate the anti-cancer activities of the extract in addition to the inhibition of cell cycle progression or induction of apoptosis^59^. Therefore, the cytotoxic effect of the AMM is due to the up-regulation of *Bax* mRNA expression, and the increase in DNA fragmentation, both of which induce an apoptotic pathway^60^. The molecular docking results confirmed these observations, indicating that the AMM is a potent anti-cancer candidate capable of activating the apoptotic pathway by inducing apoptotic cell death through increased expression of *Bax* mRNA and down-regulating the expression of *p53* and *Bcl-2*, which is well-known as a key regulator of outer mitochondrial membrane permeability. Similar results were previously reported, which demonstrated an increase in apoptotic proteins *Bcl-2* in HCT-116 cell lines, implying apoptotic cell death and supporting the idea that cell death is induced by an apoptotic pathway to produce anticancer activities^61^. The findings from the cell cycle arrest analysis demonstrated that treatment of HCT-116 colon cancer cells with AMM extract induced arrest at the G0/G1 phase. This phase corresponds to non-proliferating cells characterized by fragmented DNA, which are destined to undergo apoptosis. Such accumulation reflects DNA fragmentation and the formation of apoptotic bodies. Notably, compounds exhibiting anti-proliferative properties, regardless of their direct cytotoxicity, typically cause a significant increase in the proportion of cells in this phase^62^.

## Conclusion

HPLC analysis revealed that AM extracts are rich in total polyphenols, with kaempferol and taxifolin identified as the predominant compounds in AMM and AME extracts, respectively, and gallic acid prevailing in AMA. Among these, AMM exhibited the most potent antioxidant activity (IC₅₀ = 24 µg/mL) and pronounced anticancer effects against HCT-116 (IC₅₀ = 0.38 µg/mL) and HUH-7 (IC₅₀ = 21.95 µg/mL) cell lines, with minimal cytotoxicity toward normal BJ-1 cells (IC₅₀ = 13.05 µg/mL). Notably, AMM displayed strong selectivity toward HCT-116 cells, evidenced by its low IC₅₀ value and a high selectivity index (SI = ≥ 34.5), surpassing that observed for other extracts and the reference drug doxorubicin. These results position AMM as a promising natural candidate for anticancer therapies, especially colorectal cancer, warranting further in vivo validation.

## Materials and methods

### Plant material

The leaves of *Artemisia monosperma* were collected prior to the flowering period from wild populations located in Saint Catherine, South Sinai, Egypt (geographical coordinates 28°33′N 33°56′E) in the South Sinai Governorate, situated at the foot of Mount Sinai (Jebel Musa) and Mount Horeb (Ras Sufsafeh), at an altitude of approximately 1,500 to 1,600 m (around 5,000 to 5,250 feet) above sea level. Collection followed Egyptian Ministry of Agriculture guidelines (permit no. 550/2021) and applicable CITES regulations. The plant species was authenticated by the Plant Science Department at the National Research Centre (NRC), Cairo, Egypt. A voucher specimen (AM/SC0081/21) was deposited in the NRC Herbarium for future reference. *Artemisia monosperma* is not currently protected by major organizations such as the International Union for Conservation of Nature (IUCN), nor is it subject to trade restrictions under the Convention on International Trade in Endangered Species of Wild Fauna and Flora (CITES). All relevant local, national, and international regulations protecting this species and its habitat were strictly adhered to. All necessary permits and approvals were legally obtained from the competent authorities prior to collection. Furthermore, the research protocol received approval from the Institutional Ethics Committee of the National Research Centre, ensuring compliance with ethical standards in biodiversity conservation and sustainable use. Collection procedures were designed to minimize any potential impact on the species and its ecosystems, thus supporting ongoing conservation efforts in the region.

### Chemicals

Doxorubicin, 2,2-diphenyl-1-picrylhydrazyl (DPPH, Cat # 1898-66-4), Butylated hydroxyl anisol (BHA, Cat # 128-37-0), DMEM-F12 medium (Cat # 11320033), MTT (Cat # 475989), DMSO (Cat # 67–68-5), butylated hydroxytoluene (BHT, Cat # 128-37-0), potassium hexacyanoferrate (Cat # 104973); trichloroacetic acid (TCAA, Cat # 822342); FeCl_3_ (Cat # 7705-08-0), and other chemicals and reagents were purchased from Sigma Aldrich (MO, USA). Fetal bovine serum (Cat # A3160401) was obtained from Fisher Scientific UK Ltd. (Loughborough, UK). TRIzol reagent (Cat # 15596026) was obtained from Invitrogen (USA), RNase-free DNase kit (Cat # EN0525) was supplied by Promega Corporation (Madison, WI, USA) RT PreMix Kit (Cat # K1621) was purchased from Thermo Fisher Scientific. However, the SYBR Green PCR Master Mix (Cat # HY-K0501) was provided by Applied Biosystems (Foster City CA, USA). All the solvents used in this study were analytical grade.

### Plant extraction

The dried leaves of *A. monosperma* were extracted with different solvents: 70% methanol (AMM), 70% ethanol (AME), or aqueous (AMA) at room temperature under agitation for 36 h, and the solid-to-solvent ratio was 1:10. The extracts were filtered and evaporated under reduced pressure, and the final residue extracts were kept at 4 °C until further use.

### Determination of polyphenols by HPLC

The total polyphenols of the three extracts were determined using an Agilent 1260 series HPLC certified to ISO/IEC 17,025/2017. The separation was conducted using the Eclipse C_18_ column (4.6 mm x 250 mm i.d., 5 μm). The mobile phase consisted of water (A) and 0.05% trifluoroacetic acid in acetonitrile (B) at a flow rate of 1 ml/min and was programmed in a linear gradient as follows: 0 min (82% A); 0–5 min (80% A); 5–8 min (60% A); 8–12 min (60% A); 12–15 min (82% A) and 15–16 min (82% A). The multi-wavelength detector was monitored at 280 nm. The injection volume was 10 µl for each of the sample solutions. The column temperature was maintained at 35 °C. The data are presented as absolute concentrations, determined by quantifying individual compounds using calibration curves generated from authentic standards.

### Determination of antioxidant activity

#### DPPH assay

The antioxidant activity of *A. monosperma* extracts was assessed by the DPPH (2.2-diphenyl-1-picrylhydrazyl) free radical scavenging assay^63^. Briefly, 0.1 mM DPPH in methanol was prepared, and then 2.4 ml DPPH solution was mixed with a volume of 1.6 ml of different concentrations (25–150 µg/ml) of each extract (AMM, AME, and AMA extracts). The reaction mixture was mixed thoroughly and left in the dark at room temperature for 30 min. The transformation between the oxidized (initial violet) and reduced (yellow end-product) form of DPPH was followed by recording the absorbance decrease at 517 nm against a blank, i.e. without DPPH with UV-Vis Shimadzu (UV-1601, PC) spectrophotometer using 10 mm polystyrene. Methanol was used to zero the spectrophotometer, and the measurements were performed in triplicate. The radical scavenging activity was expressed as percentage inhibition of DPPH using the following formula:

$$\%\: \mathrm{Inhibition} = [(\text{A control} \:-\: \text{A treatment/A control})] \:\:\mathrm{X}\:\: 100$$

Where: A _control_: is the absorbance of the control; A _treatment_: is the absorbance of the treatments. Butylated hydroxyl anisol (BHA) was used as the positive control. Then % of inhibition was plotted against concentration, and from the graph, IC_50_ was calculated.

### Ferric reducing antioxidant power (FRAP)

The determination of the reducing power of *Artemisia sp* extracts was determined using the standard method of Umamaheswari and Chatterjee^64^. Different concentrations of BHA (150–25 µg/ml) were prepared and mixed with 2.5 ml of 0.2 M phosphate buffer solution (pH 6.6) and 2.5 ml of 1.0% potassium hexacyanoferrate [K_3_Fe (CN)_6_]. The reaction mixture was incubated in a water bath at for 20 mi at 50 °C. Then 2.5 ml of TCAA (10%) was added to terminate the reaction. The upper layer of the mixture was separated and mixed with 5.0 ml of distilled water and 0.5 ml of FeCl_3_ solution (0.1%). The reaction mixture was incubated for 10 min at room temperature to get a blue color solution and the absorbance at 700 nm was measured against a blank solution. In the same way, each extract (ethanol, methanol, and aqueous extracts) at different concentrations (150–25 µg/mL) was prepared, the absorbance was measured, and their activities were evaluated using the following Eq. (1):

1 $$\%\:\: \text{Reducing power} = \text{A sample}\:\mathrm{-}\: \text{A blank}/\text{A sample}\:\: {\times}\:\: 100$$

Where A _sample_: is the absorbance of the sample and A blank: is the absorbance of the blank.

All tests were performed in triplicate, the graph was plotted with the average of the three determinations, and IC_50_ was calculated from the graph.

### Cytotoxic activity

#### Cell culture

The human colorectal cancer cell line (HCT-116), human hepatoma cell line (HUH-7), and skin fibroblast (BJ-1) normal cell line were obtained from American Type Culture Collection via Professor Stig Linder, Department of Oncology and Pathology, Karolinska Institute (Stockholm, Sweden). All the cell lines were maintained in DMEM-F12 media supplemented with 10% fetal bovine serum at 37 °C in 5% CO_2_ and 95% humidity. The cells were sub-cultured using trypsin verse 0.15%. The anticancer activity was conducted at the Bioassay-Cell Culture Laboratory, National Research Centre, Cairo, Egypt, for HUH-7, HCT-116, and BJ-1 cell lines in vitro.

### Cytotoxicity on cancer monolayers

The cytotoxic assay on cancer cell lines was performed using the procedures of Thabrew et al.^65^, and El-Menshawi et al.^66^, with minor modifications. All of the following procedures were performed in a sterile environment with a laminar flow cabinet of bio-safety class II (Baker, SG403INT, and Sanford, ME, USA). Briefly, 20,000 cells per well for HCT-116, HUH-7, and BJ-1 cell lines were seeded for 24 h in 96 well plates, then the medium was changed to fresh medium, and the cells were treated with 100 µg/ml final concentration of the tested extracts in triplicates for 48 h. Doxorubicin (100 µM) was used as the positive control and DMSO (0.5%) was used as the negative control. The cell viability was determined using the MTT (3-(4, 5-dimethylthiazol-2-yl)−2,5-diphenyltetrazolium bromide) assay as described previously^67^.

The percent cytotoxicity was estimated using the following Eq:

$$\%\: \mathrm{cytotoxicity}\:=\: [1-(\mathrm{AV}_\mathrm{x}/\mathrm{AVNC})] \:\mathrm{x}\: 100$$

Where AV: is average, X is the absorbance of the sample well, and NC is the absorbance of negative control measured at 595 nm with reference at 690 nm.

### Determination of IC_50_ values

The plant extracts that shown strong cytotoxicity on different tested cell lines were selected for dose-response studies at different doses. The final tested concentrations were 100, 50, 25, 12.5, 6.25 µg/ml, and up to 0.78 µg/ml, in triplicates. The IC_50_ values were calculated using the concentration-response curve fit to the non-linear regression model using GraphPad Prism^®^ v6.0 software (GraphPad Software Inc., San Diego, CA, USA).

### Selectivity index (SI)

The selectivity index (SI) indicates the cytotoxic selectivity (i.e., safety) of different extracts against cancer cells (HCT-116, HUH-7) versus normal cells (BJ-1).

SI = IC_**50**_ of extract in the normal cell line **/** IC_**50**_ of the same extract in the cancer cell line.

The higher the SI value, the higher the safety of the extract. Extracts possessing SI value > 2 are considered safe^68^.

### Gene expression analysis for apoptotic-related genes

The gene expression analysis was conducted to determine the effect of the AMM extract on the apoptotic-related genes of HCT-116 cell lines, which produced the best results in the cytotoxicity study. In this assay, total RNA extraction, cDNA synthesis, and qRT-PCR were conducted as previously described^69^. Briefly, RNA samples were isolated using TRIzol reagent RNA, and the purity was assessed by the absorbance ratio at 260 nm and 280 nm. cDNA was prepared from samples of 1 g of RNA with SuperScript II reverse transcriptase according to the manufacturer’s protocol. Real-time PCR reactions were run on a Strata gene Mx3005P Real-Time PCR System (Agilent Technologies). The SYBR green qPCR Master Mix kit was used for qPCR analysis. One g of cDNA, 10 µM of forward and reverse primers, 10 µl TOPreal™qPCR 2× PreMIX (SYBR Green with low ROX) (Enzynomics), and DNAse-free water were used in a 20-µl reaction volume. GAPDH and β-actin were used as housekeeping genes. The primers sequences are summarized in Table 5. Each specimen was repeated four times. Finally, the fold change was calculated using the comparative delta-delta CT method (2−∆∆Ct)^75^.

**Table 5:** Details giving primer sequences for the genes amplified.

List of genes	Primer sequences (5′ to 3′)	References	Product Length(bp)
*BAX*	F: 5′-ATCCAGGATCGAGCAGGGCG − 3′ R: 5′-GGTTCTGATCAGTTCCGGCA-3′	^70^	315
*BCL-2*	F: 5′-CACAAGAGGCCAAGGCTACCT–3′ R: 5′-CAGGAAAGCAGGAAGTCTCAA-3′	^71^	158
*p53*	5′-TTGCCGTCCCAAGCAATGGA-3′ 5′-TCTGGGAAGGGACAGAAGATG-3′	^72^	193
*GAPDH*	5′-CAAGGTCATCCATGACAACTTTG-3′ 5′-GTCCACCACCCTGTTGCTGTAG-3′	^73^	496
*β-actin*	F: 5′-CCACCATGTACCCAGGCATT’−3′ R: 5′-CGGACTCATCGTACTCCTGC-3′	^74^	189

### Effect of the AMM extract on DNA Damage in HCT-116 cell lines

The apoptosis was evaluated by two different techniques that included diphenylamine (DPA) assay and DNA laddering assay. In the DPA assay, HCT-116 cell lines were treated with the AMM extract *(*0.38 µg/ml for 24 h) using diphenylamine (DPA) reagent, which binds to deoxyribose^76^. The optical density was measured at 600 nm and the proportion of fragmented DNA was calculated using the formula:

$$\text{DNA fragmentation} (\%) = [\mathrm{S/S+P})] \:\mathrm{X}\: 100$$

Where the S: fragmented DNA; and the P: intact DNA fractions.

However, in the DNA Laddering assay, genomic DNA was extracted from HCT-116 cell lines following treatment with the AMM extract. Electrophoresis was performed in 2% agarose gel with ethidium bromide staining. Following that, the DNA in the gel was visualized under UV and photographed using a digital camera.

#### Cell Cycle analysis by FACS Melody flow cytometer

The assay was performed following the method described by Abdelazeem et al.^77^, with minor modifications. HCT-116 cells were seeded in 6-well plates at a density of 1 × 10^6 cells per well and then incubated with the AMM extract for 48 h. After incubation, the cells were harvested, resuspended in 0.5 mL of 1× DPBS, aspirated multiple times, and subsequently fixed with 70% ethanol on ice for 2 h.

#### Cell cycle analysis by flow cytometry

The cell cycle was assessed using the BD Cycletest Plus DNA Kit (BD Biosciences, Catalog No. 340242) following the manufacturer’s protocol. After initial processing, cells were centrifuged for 5 min at 300 × g. The resulting pellets were resuspended in 5 ml of 1× DPBS, gently mixed for 30 s, and centrifuged again at 300 × g for 5 min. Cells were then resuspended in 1 ml of PI staining solution and incubated in the dark at room temperature for 30 min. Subsequently, the samples were analyzed using the BD Cycletest Plus DNA kit reagents along with BD CellFIT software on a BD FACScan flow cytometer equipped with DDM. The proportions of cells in the G0/G1, S, and G2/M phases were determined using BD CellFIT software^78^.

### Molecular docking

Molecular docking studies of the major bioactive constituents in the AMM extract as well as the anti-apoptotic proteins *Bcl-2* and *p53* were performed using PyRx tools Autodock Vina (version 1.1.2) as described by Dallakyan and Olson^79^. The crystal structures of *Bcl-2* (PDB ID: 2O2F) complexed with the inhibitor (LI0) and *p53* (PDB ID: 1TTV) complexed with the inhibitor (IMY) were obtained from the protein data bank at https://www.rcsb.org/structure/2O2F and https://www.rcsb.org/structure/1TTV, respectively (access on 11 May 10, 2023).

The water molecules and native ligands were removed from the protein using VEGA ZZ 2.3.2 tool followed by the addition of polar hydrogen and Kollman charges and finally converted to PDBQT format by Autodock Vina tools. All the molecules were created with ChemDraw ultra 10.0 and saved as mol files, which were then protonated, minimized, and converted to pdb files by Open Babel software. The resulting pdb file was submitted to Autodock Vina tools for several torsion settings and pdbqt file creation.

AutoGrid was combined with a grid box to create the grid map. Each compound generated 10 docking poses at the active pocket of AKT1, which were then ranked based on binding energy. The pose with the lowest binding energy and 0 Å root-mean-square deviations (RMSD) was considered to be the best fit for the receptor. The molecular interactions and binding mechanisms of the top postures were visually examined using BIOVIA Discovery Studio 2021.

### Statistical analysis

Statistical analyses were performed using SPSS version 11.0. Differences among treatment groups were assessed via one-way ANOVA followed by Tukey’s post-hoc test. Data are presented as mean ± standard error (SE), with significance set at $\:P<0.05$.

## Electronic Supplementary Material

Below is the link to the electronic supplementary material.

Supplementary Material 1

Supplementary Material 2

## Data Availability

The datasets generated during and/or analyzed during the current study are available from the corresponding author on reasonable request.

## Abbreviations

*A. Abla Aso*: *Artemisia Abla Asso*
*A. annua L.*: *Artemisia annua L*
*A. monosperma*: *Artemisia monosperma Delile*
*A. vulgaris L*.: *Artemisia vulgaris *L
*AMA*: *Artemisia monosperma aqueous leaf extract*
*AME*: *Artemisia monosperma ethanolic leaf extract*
*AMM*: *Artemisia monosperma methanolic leaf extract*
ANOVA: Analysis of Variance
ATCC: American Type Culture Collection
Bax: Bcl-2 Associated X-protein
Bcl-2: B-cell lymphoma 2
BHA: Butylated hydroxyl anisol
BHT: Butylated hydroxytoluene
BJ-1: Normal human fibroblast
cDNA: Copy Deoxyribonucleic Acid
CRC: Colorectal cancer
DMEM-F12: Ham’s F12 basic
DMEM-F12 medium: Dulbecco’s Modified Eagle Medium/Nutrient Mixture F-12
DMSO: Dimethyl sulfoxide
DNA: Deoxyribonucleic Acid
DNAse: Deoxyribonuclease
DPA: Diphenylamine
DPPH: Doxorubicin, 2,2-diphenyl-1-picrylhydrazyl
FAS: Fatty acid synthase
GAPDH: Glyceraldehyde-3-phosphate dehydrogenase
HCC: Hepatocellular carcinoma
HCT-116: Human colorectal carcinoma
HCV: Hepatitis C virus
*HPLC*: High-Performance Liquid Chromatography
HUH-7: Hepatocellular carcinoma
IC_50_: Half-maximal inhibitory concentration
IkB: Immunome Knowledge Base
mRNA: Messenger RNA
mTOR: Mammalian target of rapamycin
MTT: 2-(4,5-dimethyl-2-thiazolyl)-3,5-diphenyl-2 H-tetrazolium, monobromide
NF-kB: Nuclear factor kappa B
p53: Tumor protein p53
PARP: Poly adenosine diphosphate-ribose polymerase
PI3K/AKT: Phosphoinositide-3-kinase–protein kinase B/Akt
qRT-PCR: Quantitative reverse transcription polymerase chain reaction
RNA: Ribonucleic acid
RT: Real-time
SI: Selectivity Index
STAT3: Signal transducer and activator of transcription 3
TNF: Tumor necrosis factor
UV: Ultraviolet
XIAP: X-linked inhibitor of apoptosis protein
