# Supplementary material for: Molecular docking of polyphenols and screening of antioxidant and anticancer activity of Artemisia monosperma leaf extracts in human cancer cells
Source: Sci Rep. 2026 May 2;16:14043. doi: 10.1038/s41598-026-49276-7 (PMC13135504; doi:10.1038/s41598-026-49276-7)
Supplement: Supplementary file 1 — Supplementary Material 1 [file 41598_2026_49276_MOESM1_ESM.docx]

**Supporting information**


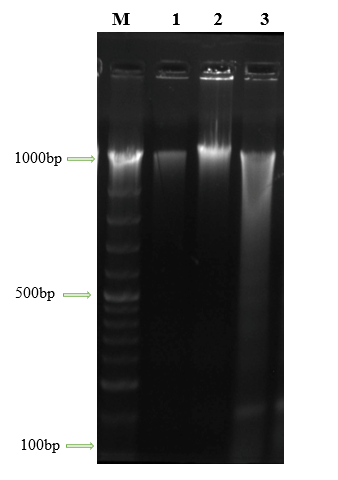


**Figure 1S.** Agarose gel electrophoresis (2%) of the DNA extracted of HCT-116 cell lines. Lane M 100 DNA marker, lane 1: Control cells, lane 2: control cell treated with 0.5 % DMSO, and lane 3: cells treated with AMM extract of *A. monosperma*


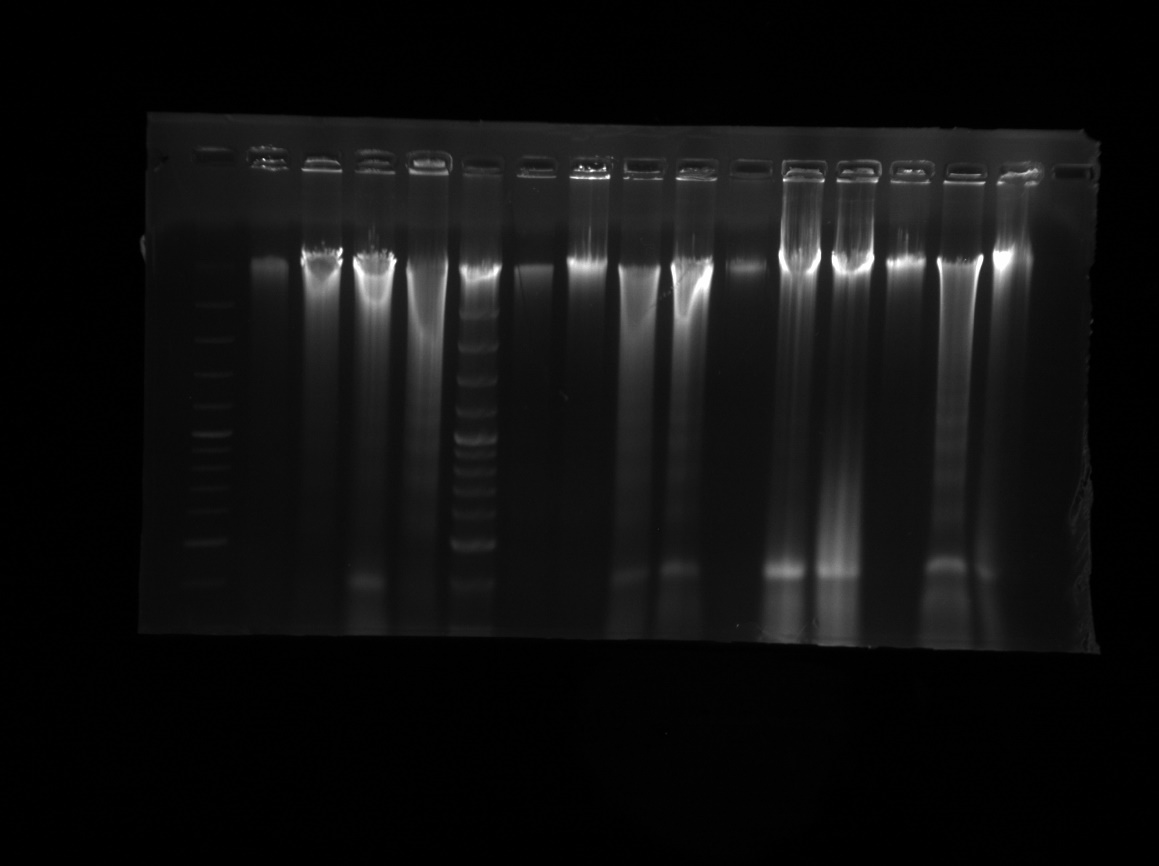


**Figure 1S.** Original agarose gel electrophoresis (2%) image showing DNA fragmentation in HCT-116 cell lines. The full gel with visible edges is presented. Lane M: 100 bp DNA marker; Lane 1: Control cells; Lane 2: Control cells treated with 0.5% DMSO; Lane 3: Cells treated with AMM extract of A. monosperma.

**Important clarifications:**

1. This figure represents agarose gel electrophoresis (2%) for DNA fragmentation analysis, not a Western blot membrane. Therefore, there are no membranes or hybridization steps involved.
2. The full gel edges are now clearly visible in the revised figure, as requested.
3. The gel was not cut prior to any processing; the original image has been provided as-is, with only relevant lanes (M, 1, 2, and 3) included to maintain focus on the experimental samples described in the study.

**The reason for choosing these proteins in case of any questions from the reviewers (not to be included in the manuscript)**

***For discussion (P53)***

MDM2 is a regulator of cell growth processes that acts by binding to the tumor suppressor protein p53 and ultimately restraining its activity. While inactivation of p53 by mutation is commonly observed in human cancers, a substantial percentage of tumors express wild type p53. In many of these cases, MDM2 is overexpressed, and it is believed that suppression of MDM2 activity could yield therapeutic benefits. Therefore, we have been focusing on the p53-MDM2 interaction as the basis of a drug discovery program and have been able to develop a series of small molecule inhibitors. We herein report a high-resolution NMR structure of a complex between the p53-binding domain of MDM2 and one of these inhibitors. The form of MDM2 utilized was an engineered hybrid between the human and Xenopus sequences, which provided a favorable combination of relevancy and stability. The inhibitor is found to bind in the same site as does a highly potent peptide fragment of p53. The inhibitor is able to successfully mimic the peptide by duplicating interactions in three subpockets normally made by amino acid sidechains, and by utilizing a scaffold that presents substituents with rigidity and spatial orientation comparable to that provided by the alpha helical backbone of the peptide. The structure also suggests opportunities for modifying the inhibitor to increase its potency.

**For BCL2**

Overexpression of the antiapototic proteins Bcl-2 and Bcl-xL provides a common mechanism through which cancer cells gain a survival advantage and become resistant to conventional chemotherapy. Inhibition of these prosurvival proteins is an attractive strategy for cancer therapy. We recently described the discovery of a selective Bcl-xL antagonist that potentiates the antitumor activity of chemotherapy and radiation. Here we describe the use of structure-guided design to exploit a deep hydrophobic binding pocket on the surface of these proteins to develop the first dual, subnanomolar inhibitors of Bcl-xL and Bcl-2. This study culminated in the identification of 2, which exhibited EC50 values of 8 nM and 30 nM in Bcl-2 and Bcl-xL dependent cells, respectively. Compound 2 demonstrated single agent efficacy against human follicular lymphoma cell lines that overexpress Bcl-2, and efficacy in a murine xenograft model of lymphoma when given both as a single agent and in combination with etoposide.
