# Supplementary figures and images for: Molecular docking of polyphenols and screening of antioxidant and anticancer activity of Artemisia monosperma leaf extracts in human cancer cells
Source: Sci Rep. 2026 May 2;16:14043. doi: 10.1038/s41598-026-49276-7 (PMC13135504; doi:10.1038/s41598-026-49276-7)

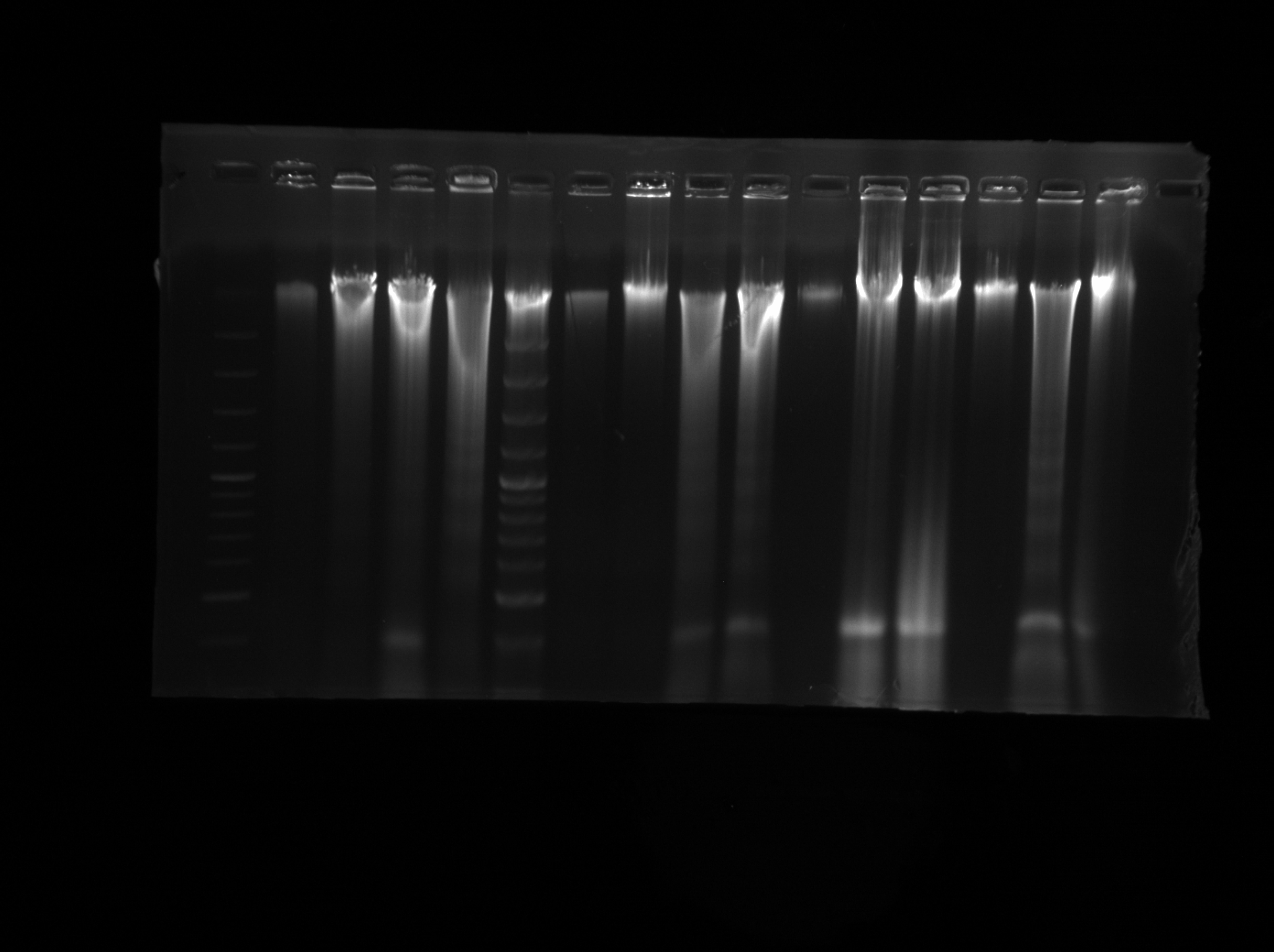

Supplement: Supplementary file 2 — Supplementary Material 2 [file 41598_2026_49276_MOESM2_ESM.jpeg]
